# Role of Urinary Matrix Metalloproteinase-7 (MMP-7) as an Early Marker of Renal Dysfunction in Diabetic Individuals: A Cross-Sectional Study

**DOI:** 10.7759/cureus.66392

**Published:** 2024-08-07

**Authors:** Rajlaxmi Sarangi, Debadyuti Sahu, Nikunj Kishore Rout, Krishna Padarabinda Tripathy, Saurav Patra, Jyotirmayee Bahinipati, Jyoti Prakash Sahoo

**Affiliations:** 1 Biochemistry, Kalinga Institute of Medical Sciences, Bhubaneswar, IND; 2 Nephrology, Kalinga Institute of Medical Sciences, Pradyumna Bal Memorial Hospital, Bhubaneswar, IND; 3 General Medicine, Kalinga Institute of Medical Sciences, Bhubaneswar, IND; 4 Pharmacology, Kalinga Institute of Medical Sciences, Bhubaneswar, IND

**Keywords:** glycemic status, gender variation, types 2 diabetes, estimated glomerular filtrate rate, matrix metalloproteinase, urine albumin creatinine ratio, severe renal impairment, diabetic kidney disease (dkd)

## Abstract

Background and objectives: The matrix metalloproteinase 7 (MMP-7) level gets heightened in the urine samples of diabetic individuals with impaired renal function. Renal biopsy is seldom offered because of its invasive nature. These concerns spurred the investigation of relationships between urine MMP-7 levels and the renal function of diabetic individuals. Studies exploring this aspect are scarce. We aimed to evaluate the glycemic and renal parameters of female and male individuals with or without type 2 diabetes mellitus (T2DM) or kidney disease. We also assessed the correlation of urine MMP-7 with various parameters.

Methods: This prospective, analytical, cross-sectional study was conducted at Kalinga Institute of Medical Sciences (KIMS), Bhubaneswar, India, from February 2020 to January 2023. Female and male individuals 18-85 years of age diagnosed with either T2DM, hypertension, or kidney disease were assessed for their glycemic indices and renal parameters. Those with both renal disease and T2DM were placed in group A. The diabetic individuals without kidney disease constituted group B. People in group C had neither kidney disease nor T2DM. Patients in group D had kidney disease but were not diabetics. The parameters of the male and female participants in each of the four groups were assessed and compared, including: age, body mass index (BMI), fasting blood sugar (FBS), glycosylated hemoglobin (HbA1c), serum urea, serum creatinine, estimated glomerular filtration rate (eGFR), urine albumin, urine creatinine, urine albumin-creatinine ratio (ACR), serum sodium, serum potassium, and urine MMP-7 levels. Furthermore, we correlated urine MMP-7 with all these traits. We used R software (version 4.4.0, Vienna, Austria) for data analysis.

Results: Two hundred eighty-seven (87.5%) of the 328 individuals we screened were eligible. Of them, group A had the maximum number (94) of participants, followed by B (75), C (65), and D (53). Males comprised 60.3% (n = 173) of the study population. The median age of the participants was 52.0 (44.0-61.1) years. The intergroup variations were statistically significant (p < 0.001) owing to their glycemic status and renal function. The gender-basis comparison of FBS and HbA_1c_ yielded non-significant differences. On the contrary, assessment of the renal parameters revealed significant differences (p < 0.001) between females and males. The study population had a median urine MMP-7 level of 19.9 (1.1-50.5) µg/L. Significant associations with urine MMP-7 were found with serum creatinine (r = 0.91, p < 0.001), urine ACR (r = 0.86, p < 0.001), and eGFR (r = -0.84, p < 0.001).

Conclusion: Our study portrayed that male diabetics, in comparison to female diabetics, had greater levels of urine ACR, urine MMP-7, eGFR, and serum creatinine. Moreover, urine ACR, eGFR, and serum creatinine strongly correlated with the urine MMP-7 level.

## Introduction

A class of endopeptidases known as matrix metalloproteinase (MMP) cleaves the collagen and non-collagenous glycoproteins that make up the extracellular matrix (ECM). These enzymes are zinc- and calcium-dependent. For the functionality of their active sites and optimum tertiary structure, respectively, MMPs necessitate calcium and zinc ions, and MMP-7 is one of the most important variants [1]. Collagen IV and X, transferrin, laminin, fibronectin, plasminogen, and other constituents of the extracellular matrix (ECM) are all broken down by MMP-7. It also modulates MMP-1, MMP-2, and MMP-9 activity [1]. A major target for microvascular complications in individuals with type 2 diabetes mellitus (T2DM) is the kidney [2]. The term “diabetic nephropathy” was rechristened as diabetic kidney disease (DKD) in 2007 by the Kidney Disease Outcome Quality Initiative (KDOQI) [3]. The renin-angiotensin-aldosterone system (RAAS) and the gremlin-1 pathway, commonly referred to as the bone morphogenic protein (BMP) pathway, are dysregulated in DKD. Because these pathways are abnormal in DKD patients, there is an increased excretion of gremlin-1, MMP-7, and angiotensinogen in the urine [4,5]. The expression of kidney angiotensinogen is elevated in animal models of DKD. The concentration of gremlin-1 is also upregulated in the DKD animal models. It accelerates the development of kidney disease [4].

The first indication of DKD is abnormal ECM accumulation, which causes glomerular basement membrane (GBM) thickening. Chronic accumulation brought on by aberrant ECM turnover impairs renal function [4,6]. The final result of DKD is end-stage renal disease (ESRD), which develops from a variety of kidney damage, including microalbuminuria, proteinuria, and reduced renal function [7]. Lately, a few articles revealed a handful of instances of DKD with a reduced estimated glomerular filtration rate (eGFR) and no evidence of microalbuminuria [8-11]. Renal biopsy is a highly accurate diagnostic technique for discriminating DKD from other kidney ailments. Nevertheless, owing to its invasive nature, it is only sometimes recommended [12].

The synthesis of advanced glycation end products (AGEs) entails many mechanisms. These AGEs quicken DKD's pathogenesis [13,14]. According to recent investigations, a higher level of urine MMP-7 in DKD is linked to AGEs [15-17]. Patients with DKD have altered renal expression levels of MMP-7 because it is often expressed in the renal tubular epithelium [16,18]. Trace quantities of MMP-7 are seen in the healthy renal tubular epithelium. Nonetheless, its overexpression has been connected to several renal pathological conditions, such as DKD, hydronephrosis, and autosomal dominant polycystic kidney disease [15]. Remarkably, higher urine MMP-7 levels are linked to all-cause death in individuals with DKD [15]. Because urine MMP-7 originates in the kidneys, it is more credible than serum MMP-7 in patients with kidney disease, regardless of their glycemic status [18]. Consequently, measurement of urine MMP-7 renders a more precise portrayal of renal function than serum MMP-7. Urine creatinine levels, however, must be taken into account when determining the urine MMP-7 level [18,19].

Therefore, it is imperative to identify a noninvasive surrogate biomarker that can alert people with or without T2DM to renal fibrosis, even in the absence of elevated serum creatinine, urine albumin, or altered eGFR. Furthermore, in patients with T2DM, this biomarker can be employed as a predictive indicator for the severity of DKD. Given this, we planned this study to evaluate urine MMP-7 levels in individuals with or without renal disease and T2DM.

## Materials and methods

Between February 2020 and January 2023, this cross-sectional study was conducted. Before we commenced the study, we got ethical approval (KIIT/KIMS/IEC/211/2020) from the Institutional Ethics Committee of Kalinga Institute of Medical Sciences (KIMS), Bhubaneswar, India. Each participant provided written informed consent prior to the registration process. The research adhered to the Declaration of Helsinki, national laws, and institutional policies. The data gathered was kept confidential.

Study participants

The study comprised adult people, regardless of gender, aged between 18 and 85 years, who had been diagnosed with either renal disease, hypertension, or T2DM. Patients having a kidney transplant, those on hemodialysis, those with autoimmune diseases affecting the kidneys, those with infectious diseases affecting the kidneys, those with inherited kidney disease, those under treatment for diabetic retinopathy, those with malignancies, rheumatoid arthritis, and those who had experienced a thrombo-embolic event within the preceding six months were all excluded from the study. Nor did this study include mothers who were nursing or pregnant.

Study design and objectives

The glycemic status and renal function were utilized to categorize the participants in this cross-sectional study and gauge their clinical characteristics. For the group assortment, we did not take hypertension into account. We grouped the participants based on their clinical diagnoses of renal disease and T2DM. Those with both renal disease and T2DM were placed in group A. The diabetic individuals without kidney disease constituted group B. People in group C had neither kidney disease nor T2DM. Patients in group D had kidney disease but were not diabetics.

The objective of the study was to evaluate and compare the following characteristics of the participants in the four groups: age, body mass index (BMI), fasting blood sugar (FBS), glycosylated hemoglobin (HbA1c), serum urea, serum creatinine, eGFR, urine albumin, urine creatinine, urine albumin-creatinine ratio (ACR), serum sodium, serum potassium, and urine MMP-7 levels. Correlating the urine MMP-7 levels with the above-mentioned parameters was an additional objective.

Study procedure

Following overnight fasting for 8-12 hours, 3 ml of blood and 5 ml of the first-morning midstream urine sample were collected from each participant for quantitative estimation of various parameters with appropriate quality control. Under aseptic and antiseptic measures, 3 ml of venous blood was drawn from the medial cubital vein of all subjects. Of that sample, 1 ml of blood was preserved in a fluoride vial for the FBS estimation. Another 1 ml of blood was kept in an ethylene-diamine tetraacetic acid (EDTA) vial for HbA1c estimation. The remaining 1 ml sample was kept in a red-top vacutainer for serum separation. After the clot retraction, the serum sample was centrifuged at 3000 revolutions per minute (RPM) for 10 minutes. The quantitative measurements of serum urea and serum creatinine were done by DxC 700 AU Beckman Coulter Autoanalyzer®. HbA1c was measured quantitatively by high-performance liquid chromatography (HPLC) in Tosoh HLC-723GX®.

The 5 ml of first-morning midstream urine sample was collected in a sterile container. From that sample, 1 ml was analyzed quantitatively to evaluate urine albumin, creatinine, and ACR. The microalbumin level in the urine sample was quantified by immunoturbidimetry and urinary creatinine by Jaffe's method in the DxC 700 AU Beckman Coulter Autoanalyzer®. The urine albumin-to-creatinine (ACR) ratio was represented as mg of albumin per gram of creatinine excretion. The remaining 4 ml of urine was centrifuged at 2000-3000 RPM for 20 minutes. From that, 100 microliters of supernatant were preserved at -80°C for the quantitative estimation of MMP-7 by enzyme-linked immunosorbent assay (ELISA).

The urine MMP-7 levels were measured once a week through a RayBIO® Human MMP-7 ELISA kit (catalog number: ELH-MMP7). Except for MMP-7, the rest of the urine parameters were analyzed on the same day of collection in the central laboratory of the Biochemistry Department. The urinary MMP-7 values reported in the current study were obtained after normalization of the measured urinary MMP-7 values of each participant with their urinary creatinine values. The ratio of urine MMP-7 to urine creatinine provided the normalized urine MMP-7 values. The eGFR was quantified by using the modification of diet in renal disease (MDRD) formula [20]. Using the MDRD formula (eGFR = 175 × SCr^-1.154^ × Age^-0.203^ × 0.742 (if female) × 1.21 (if Black)), the kidney disease staging was done as follows: stage I: eGFR ≥90 mL/min/1.73 m^2^; stage II: eGFR 60-89 mL/min/1.73 m^2^; stage III: eGFR 30-59 mL/min/1.73 m^2^; stage IV: eGFR 15-29 mL/ min/1.73 m^2^; and stage V: eGFR <15 mL/min/1.73 m^2^. For this study, we considered stages IV and V as kidney disease.

Statistical analysis

We assumed mean proportions of 0.6 and 0.4 for the incidence of kidney disease in diabetic and non-diabetic individuals to determine the sample size. A two-sided alpha error of 0.05 and a beta error of 0.10 (i.e., 90% power) were obligated for 258 cases. Considering the Fleiss correction for continuity, we calculated the sample size to be 277. However, the final analysis included 287 participants (169 with diabetes in groups A and B and 118 non-diabetics in groups C and D).

Through the Shapiro-Wilk and the Kolmogorov-Smirnov tests, we verified the data distribution's normality and found it non-parametric. Frequency and proportion served as the summary statistics for qualitative data. The median and interquartile range (IQR) were adopted to portray the quantitative data. Using Pearson's chi-square test, we evaluated the qualitative data. The Kruskal-Wallis test gauged the quantitative data. For post-hoc analysis, the Bonferroni test was selected. We used the R software (version 4.4.0) [21] for data analysis and plot generation. All the statistical tests were two-tailed. Statistical significance was explained for p-values < 0.05.

## Results

We conducted this cross-sectional study from February 2020 to January 2023. A total of 328 patients were screened for this study. Thirty-four refused participation, while seven fell short of the age criteria. Two hundred eighty-seven patients were categorized into four study groups based on their glycemic status (T2DM) and renal parameters (kidney disease staging per MDRD formula). Table 1 corroborated the demographic and clinical details of the study population. Ninety-four participants with renal disease and T2DM were placed in group A. Seventy-five diabetic individuals without kidney disease constituted group B. Sixty-five subjects in group C had neither kidney disease nor T2DM. Fifty-three participants in group D had kidney disease but were not diabetics. The median age of the participants was 52.0 (44.0-61.1) years. The median eGFR was 35.7 (24.2-92.3) mL/min/1.73 m^2^. The participants had a median urine ACR of 151.2 (10.7-199.1) mg/g. The median value of normalized urine MMP-7 level was 19.9 (1.1-50.5) µg/L. Because of their group characteristics regarding the presence of T2DM and kidney disease, all of the demographic traits and clinical parameters that were compared across groups showed statistically significant differences.

**Table 1:** Demographic traits and clinical parameters of the study participants. The median with an interquartile range (IQR) was chosen to depict the continuous variables. Frequency and percentage were the measures used to display the categorical variables. The continuous and categorical variables were gauged with the Kruskal-Wallis and Chi-square (ꭓ^2^) tests, respectively. Groups A, B, C, and D entailed individuals with diabetic nephropathy, diabetes only, without diabetes and nephropathy, and nephropathy only. BMI: body mass index, eGFR: estimated glomerular filtration rate, urine ACR: ratio of urine albumin to urine creatinine, MMP-7: matrix metalloproteinase 7, normalized urine MMP-7: ratio of urine MMP-7 to urine creatinine.

Parameters	Total (n = 287)	Group A (n = 94)	Group B (n = 75)	Group C (n = 65)	Group D (n = 53)	p-value
Age (years)	52.0 (44.0-61.0)	57.5 (49.0-63.0)	47.0 (40.5-55.0)	44.0 (30.0-52.0)	56.0 (51.0-63.0)	<0.001
Age group						
<40 years	44 (15.3%)	3 (3.2%)	12 (16.0%)	29 (44.6%)	0	<0.001
40-60 years	170 (59.2%)	55 (58.5%)	53 (70.7%)	28 (43.1%)	34 (64.2%)	
>60 years	73 (25.5%)	36 (38.3%)	10 (13.3%)	8 (12.3%)	19 (35.8%)	
Males (%)	173 (60.3%)	68 (72.3%)	38 (50.7%)	35 (53.8%)	32 (60.4%)	<0.001
BMI (kg/m^2^)	24.2 (22.3-25.2)	22.1 (20.9-24.2)	24.4 (23.2-25.1)	24.5 (23.7-25.9)	24.4 (23.8-25.4)	<0.001
Urea (mg/dL)	41.0 (10.0-56.0)	58.0 (48.0-62.0)	11.0 (10.5-12.0)	9.0 (8.0-10.0)	55.0 (51.0-62.0)	<0.001
Serum creatinine (mg/dL)	1.97 (0.81-2.48)	2.65 (2.18-2.86)	0.81 (0.73-0.87)	0.81 (0.72-0.84)	2.34 (2.17-2.57)	<0.001
eGFR (mL/min/m^2^)	35.7 (24.2-92.3)	23.7 (21.5-26.1)	90.6 (84.1-96.0)	95.7 (87.8-105.9)	24.5 (23.2-29.9)	<0.001
Urine albumin (µg/dL)	3.38 (1.11-4.23)	4.83 (4.14-5.20)	1.22 (1.11-1.34)	1.04 (0.97-1.09)	3.77 (3.53-4.12)	<0.001
Urine creatinine (mg/dL)	35.8 (20.5-111.9)	24.5 (18.8-30.4)	88.9 (67.9-101.5)	173.6 (154.2-198.6)	16.9 (14.7-20.1)	<0.001
Urine ACR (mg/g)	151.2 (10.7-199.1)	163.7 (156.9-277.2)	13.7 (11.9-17.4)	6.1 (5.3-6.7)	243.4 (168.8-255.9)	<0.001
Serum Na (mg/dL)	140.0 (137.8-142.9)	140.1 (137.5-143.9)	140.2 (138.0-142.5)	140.0 (137.9-142.8)	139.8 (137.3-142.9)	<0.001
Serum K (mg/dL)	4.57 (4.07-4.79)	4.62 (4.16-4.94)	4.44 (3.95-4.65)	4.48 (4.25-4.66)	4.91 (3.77-5.10)	<0.001
Normalized urine MMP-7 levels (µg/L)	19.9 (1.1-50.5)	64.4 (51.8-89.6)	1.4 (1.1-1.8)	0.9 (0.7-1.1)	34.5 (23.5-40.4)	<0.001

**Figure 1:**
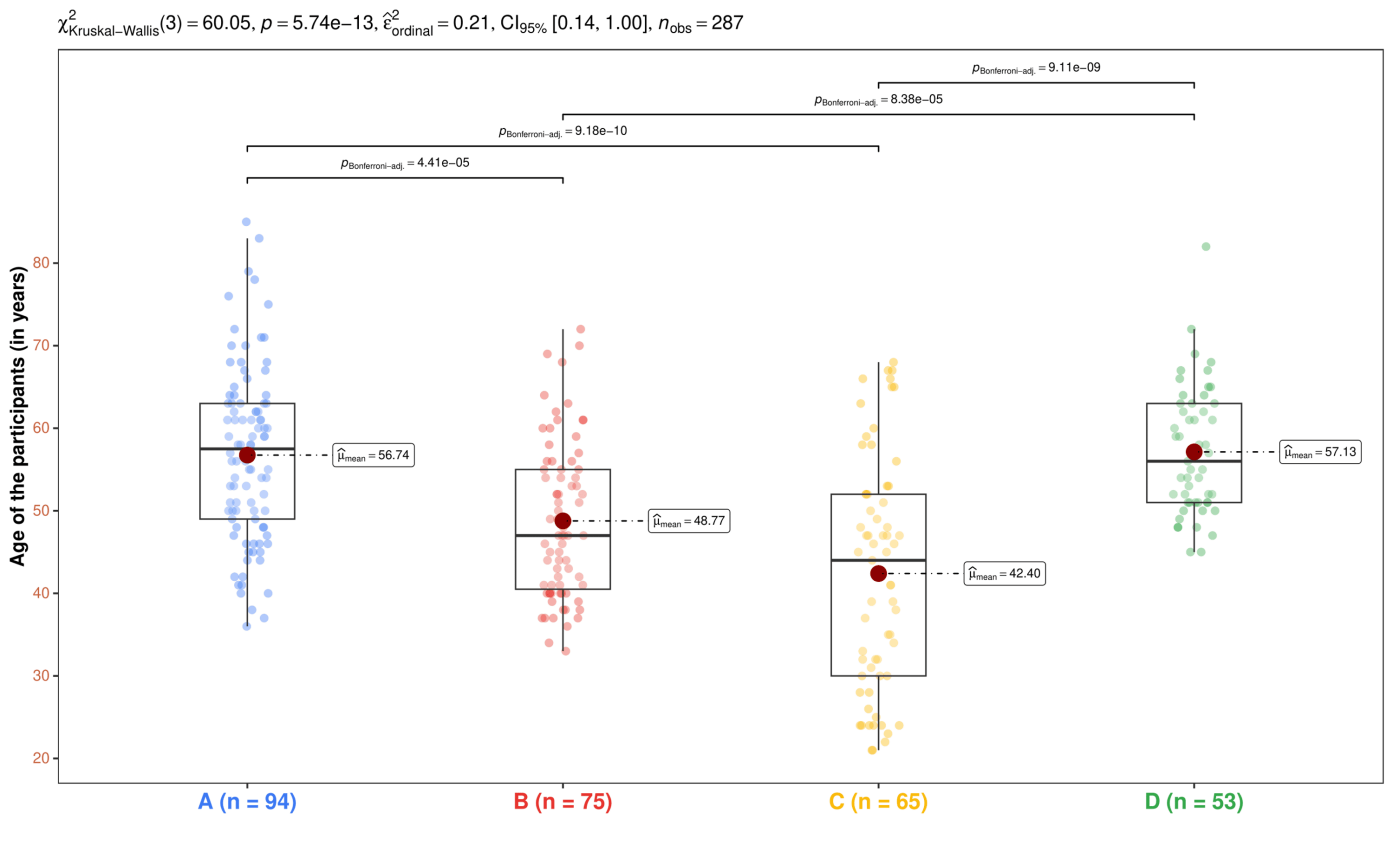
Intergroup comparison and post-hoc analysis of age. The age distributions for the four groups' subjects are displayed via the box-whisker and jitter plots. The mean values are highlighted through the red dots. The Bonferroni test was used after the Kruskal-Wallis test for the intergroup comparison. Only the statistically significant differences are illustrated. Groups A, B, C, and D entailed individuals with diabetic nephropathy, diabetes only, without diabetes and nephropathy, and nephropathy only.

In Figure 1, we display the age distribution pattern of the participants in the four study groups. The median age values for the corresponding groups were 57.5 (49.0-63.0), 47.0 (40.5-55.0), 44.0 (30.0-52.0), and 56.0 (51.0-63.0) years, respectively (p < 0.001). The differences could be attributed to the glycemic status and degree of renal impairment of the four groups’ participants. The post-hoc analysis with the Bonferroni test yielded statistically significant differences (p < 0.001) between groups A and B, A and C, B and D, and C and D. The non-significant differences in age distribution between groups A and D and B and C could be attributed to the presence or absence of diabetes mellitus in the corresponding groups.

**Figure 2:**
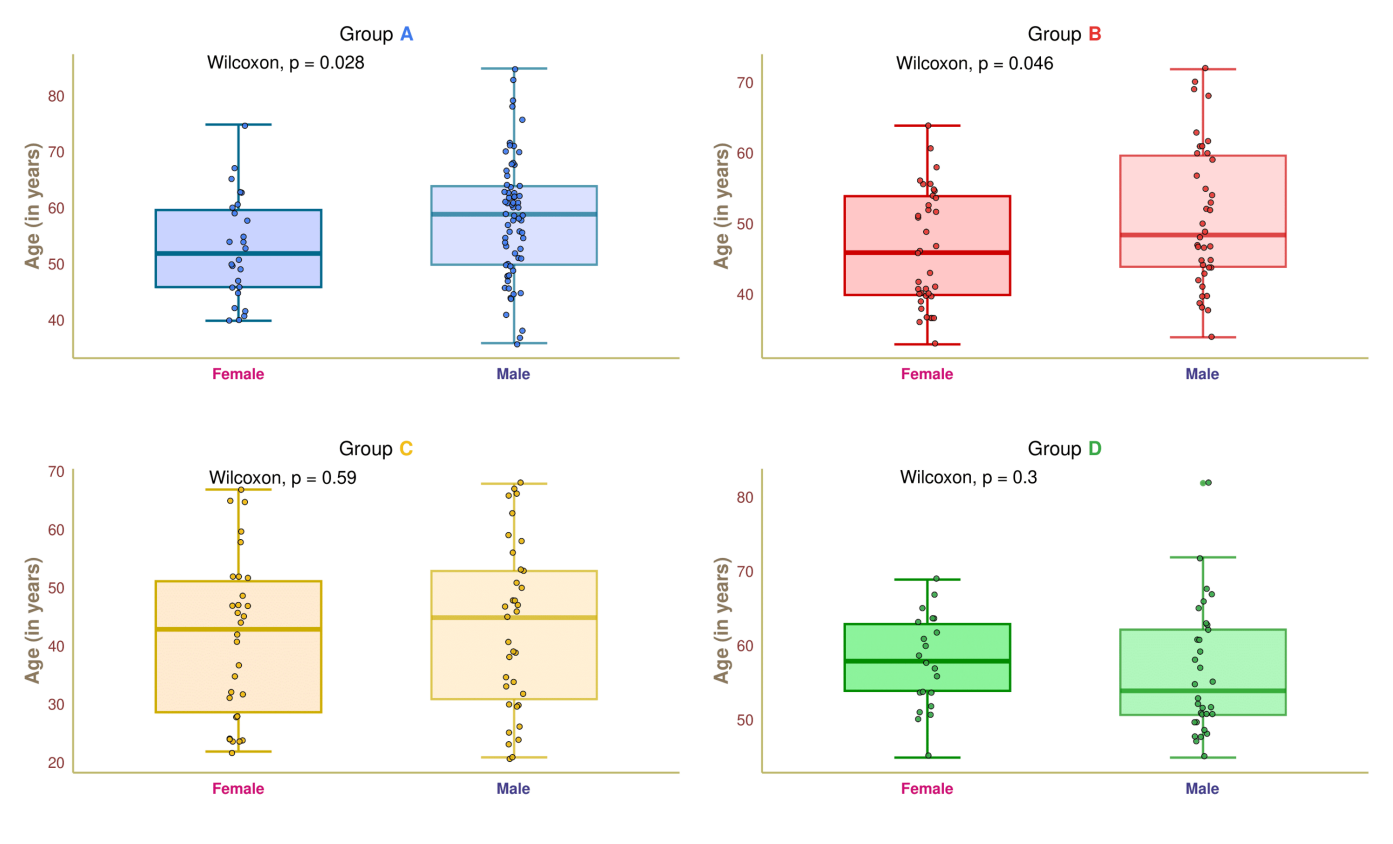
Age distribution of female and male participants. The age distributions for the four groups' female and male participants are depicted through the box-whisker and jitter plots. The Wilcoxon test was applied to gauge the differences between female and male participants. Groups A, B, C, and D entailed individuals with diabetic nephropathy, diabetes only, without diabetes and nephropathy, and nephropathy only.

Figure 2 depicts the age distribution of female and male participants in the four study groups. The median ages of group A female and male participants were 51.5 (47.0-59.5) and 59.0 (49.5-62.5) years, respectively (p = 0.028). The median ages of group B participants were 45.5 (40.0-54.5) and 47.0 (44.0-59.0) years, respectively (p = 0.046). These findings suggested that the median age of female diabetic patients was lower than that of male diabetics. The median ages of our female and male participants in group C were 43.0 (29.0-51.10) and 44.0 (30.5-52.10) years, respectively (p = 0.59). The median ages of group D participants were 58.0 (54.0-63.0) and 54.0 (51.0-62.0) years, respectively (p = 0.30).

**Figure 3:**
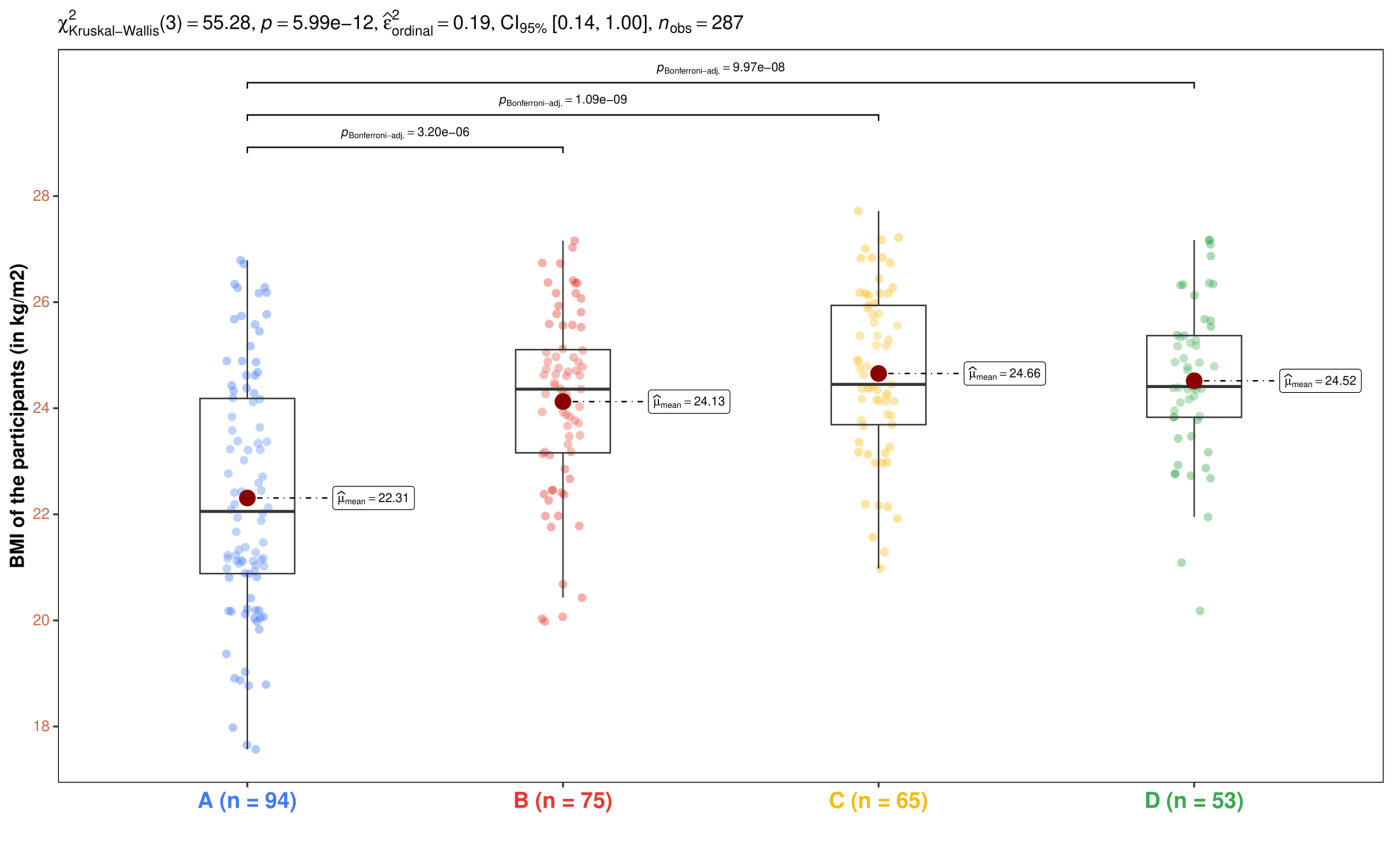
Intergroup comparison and post-hoc analysis of BMI. The four groups' subjects' BMI values (in kg/m^2^) are displayed via the box-whisker and jitter plots. The mean values are highlighted through the red dots. The Bonferroni test was used after the Kruskal-Wallis test for the intergroup comparison. Only the statistically significant differences are illustrated. Groups A, B, C, and D entailed individuals with diabetic nephropathy, diabetes only, without diabetes and nephropathy, and nephropathy only. BMI: body mass index.

In Figure 3, we portrayed the BMI values of the participants of the four study groups. The median BMI values for the corresponding groups were 22.1 (20.9-24.2), 24.4 (23.2-25.1), 24.5 (23.7-25.9), and 24.4 (23.8-25.4) kg/m^2^, respectively (p < 0.001). The differences could be attributed to the glycemic status and degree of renal impairment of the four groups' participants. The post-hoc analysis with the Bonferroni test yielded statistically significant differences (p < 0.001) between the participants of group A and the other three groups. These significant differences could be attributed to the presence of diabetes mellitus and nephropathy in the group A participants.

**Figure 4:**
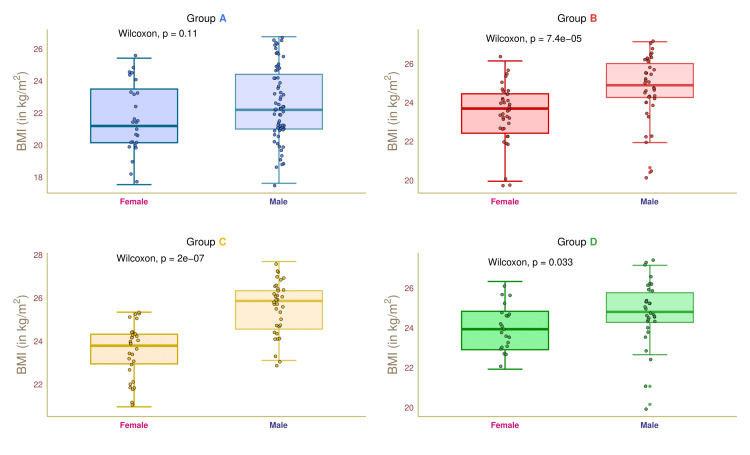
BMI of female and male participants. The BMI values for the four groups' female and male participants are depicted through the box-whisker and jitter plots. The Wilcoxon test was applied to gauge the differences between female and male participants. Groups A, B, C, and D entailed individuals with diabetic nephropathy, diabetes only, without diabetes and nephropathy, and nephropathy only.

Figure 4 depicts the BMI values of female and male participants in the four study groups. The median BMI values of group A female and male participants were 21.0 (20.0-23.5) and 22.0 (21.0-24.5) kg/m^2^, respectively (p = 0.11). The median BMI values of group B participants were 23.5 (22.5-24.5) and 25.0 (24.5-26.0) kg/m^2^, respectively (p < 0.001). The median BMI values of the female and male participants in group C were 23.8 (23.0-24.3) and 25.8 (24.5-26.3) kg/m^2^, respectively (p < 0.001). The median BMI values of group D participants were 24.0 (23.0-25.0) and 25.0 (24.5-25.8) kg/m^2^, respectively (p = 0.033). These findings suggested that female patients' median BMI was lower than male patients. However, the differences in BMI between female and male patients with diabetic nephropathy were not statistically significant.

**Figure 5:**
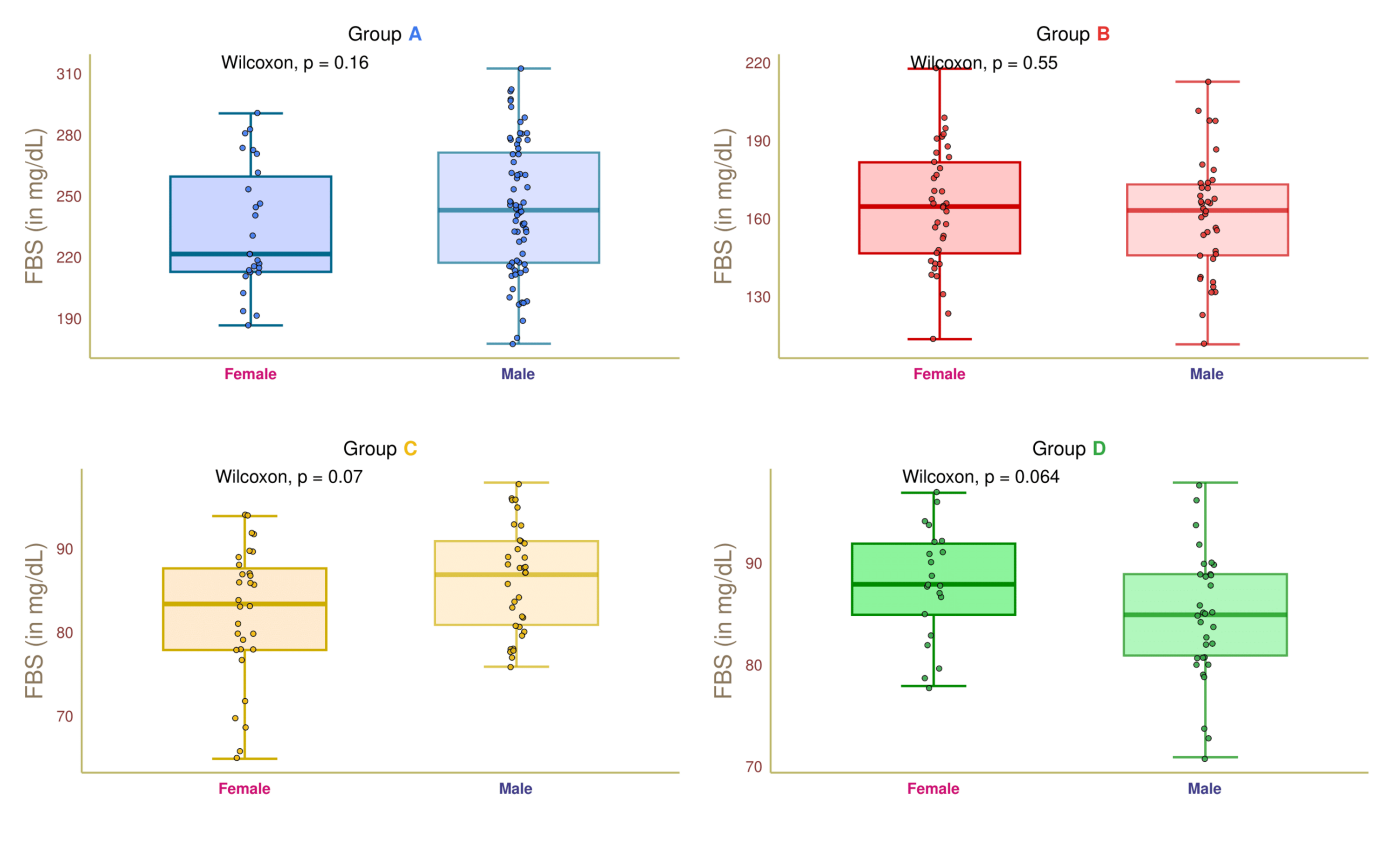
FBS of female and male participants. The box-whisker and jitter plots depict the FBS values for the four groups' female and male participants. The Wilcoxon test was applied to gauge the differences between female and male participants. Groups A, B, C, and D entailed individuals with diabetic nephropathy, diabetes only, without diabetes and nephropathy, and nephropathy only. FBS: fasting blood sugar.

Figure 5 illustrates the FBS values of female and male participants in the four study groups. The median FBS values of group A female and male participants were 221.0 (213.0-261.5) and 239.0 (219.0-271.5) mg/dL, respectively (p = 0.16). The median FBS values of female and male participants in group B were 164.5 (147.0-181.0) and 25.0 (24.5-26.0) mg/dL, respectively (p = 0.55). The median FBS values of group C participants were 84.0 (78.0-88.0) and 87.0 (80.0-90.0) mg/dL, respectively (p = 0.07). The median FBS values of group D participants were 88.0 (85.0-92.0) and 85.0 (81.0-89.0) mg/dL, respectively (p = 0.064).

**Figure 6:**
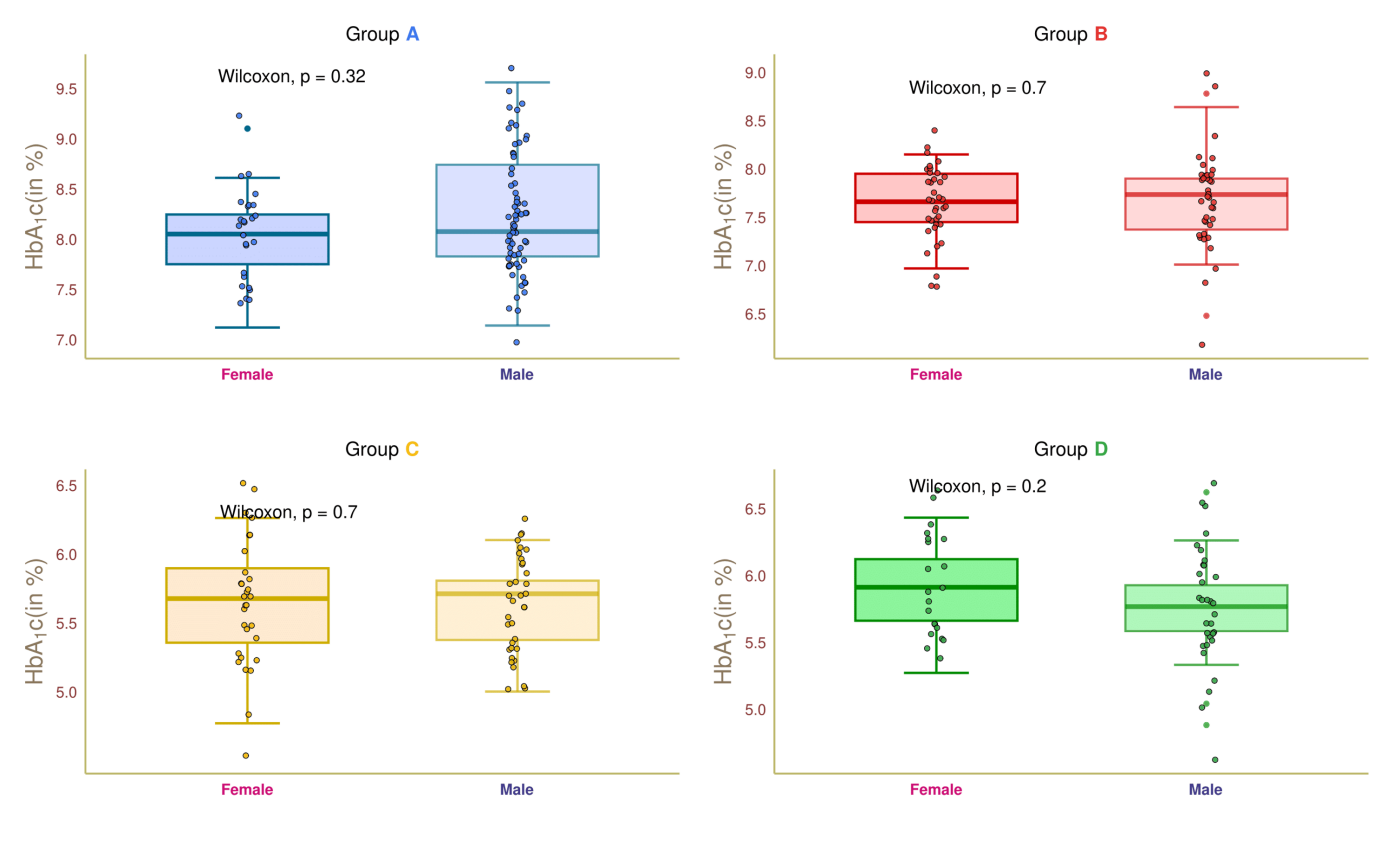
HbA1c of female and male participants. The box-whisker and jitter plots depict the HbA1c values for the four groups' female and male participants. The Wilcoxon test was applied to gauge the differences between female and male participants. Groups A, B, C, and D entailed individuals with diabetic nephropathy, diabetes only, without diabetes and nephropathy, and nephropathy only. HbA1c: glycosylated hemoglobin.

Figure 6 depicts the HbA1c values of female and male participants in the four study groups. The median HbA1c values of group A female and male participants were 8.1 (7.8-8.5) and 8.1 (7.9-8.5), respectively (p = 0.32). The median HbA1c values of group B participants were 7.6 (7.4-7.9) and 7.7 (7.3-7.8%), respectively (p = 0.7). The median HbA1c​​​​​​​ values of the female and male participants of group C were 5.7 (5.4-5.9) and 5.7 (5.4-5.8) %, respectively (p = 0.7). The group D female and male participants had median HbA1c​​​​​​​ values of 5.9 (5.6-6.1) and 5.5 (5.1-5.7%), respectively (p = 0.2). These findings suggested that median HbA1c​​​​​​​ fluctuated with the presence and severity of diabetes and nephropathy.

**Figure 7:**
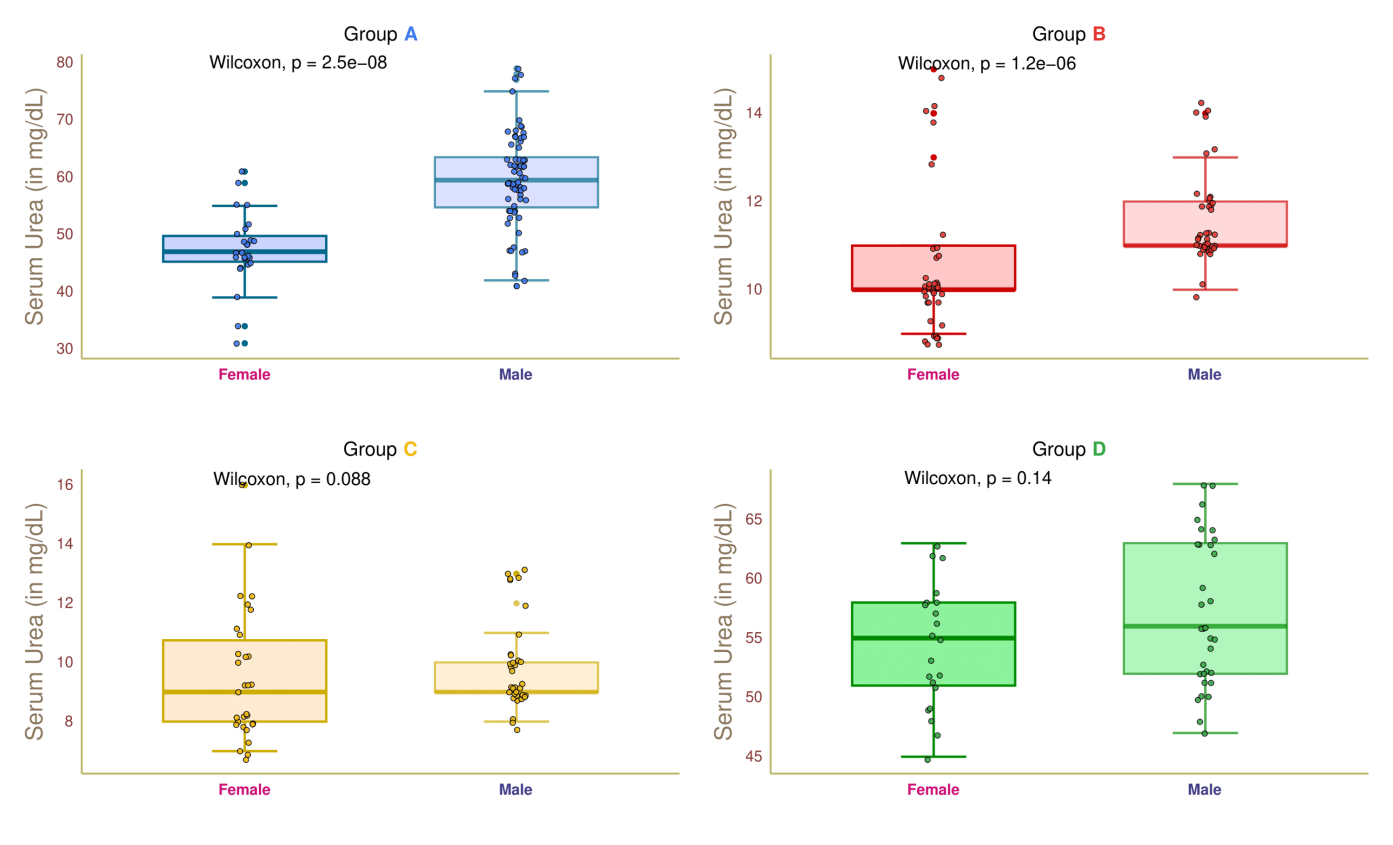
Serum urea of female and male participants. The serum urea values for the four groups' female and male participants are depicted through the box-whisker and jitter plots. The Wilcoxon test was applied to gauge the differences between female and male participants. Groups A, B, C, and D entailed individuals with diabetic nephropathy, diabetes only, without diabetes and nephropathy, and nephropathy only.

Figure 7 illustrates the serum urea values of female and male participants in the four study groups. The median serum urea values of group A female and male participants were 46.5 (45.0-49.5) and 59.0 (55.0-62.5) mg/dL, respectively (p < 0.001). The median serum urea values of female and male participants in group B were 10.0 (10.0-11.0) and 11.0 (11.0-12.0) mg/dL, respectively (p < 0.001). The median serum urea values of group C participants were 9.0 (8.0-10.8) and 9.0 (9.0-9.8) mg/dL, respectively (p = 0.088). The median serum urea values of group D participants were 55.0 (51.0-57.5) and 55.5 (51.5-62.5) mg/dL, respectively (p = 0.14). These findings suggested that diabetic males had higher urea levels than diabetic females.

**Figure 8:**
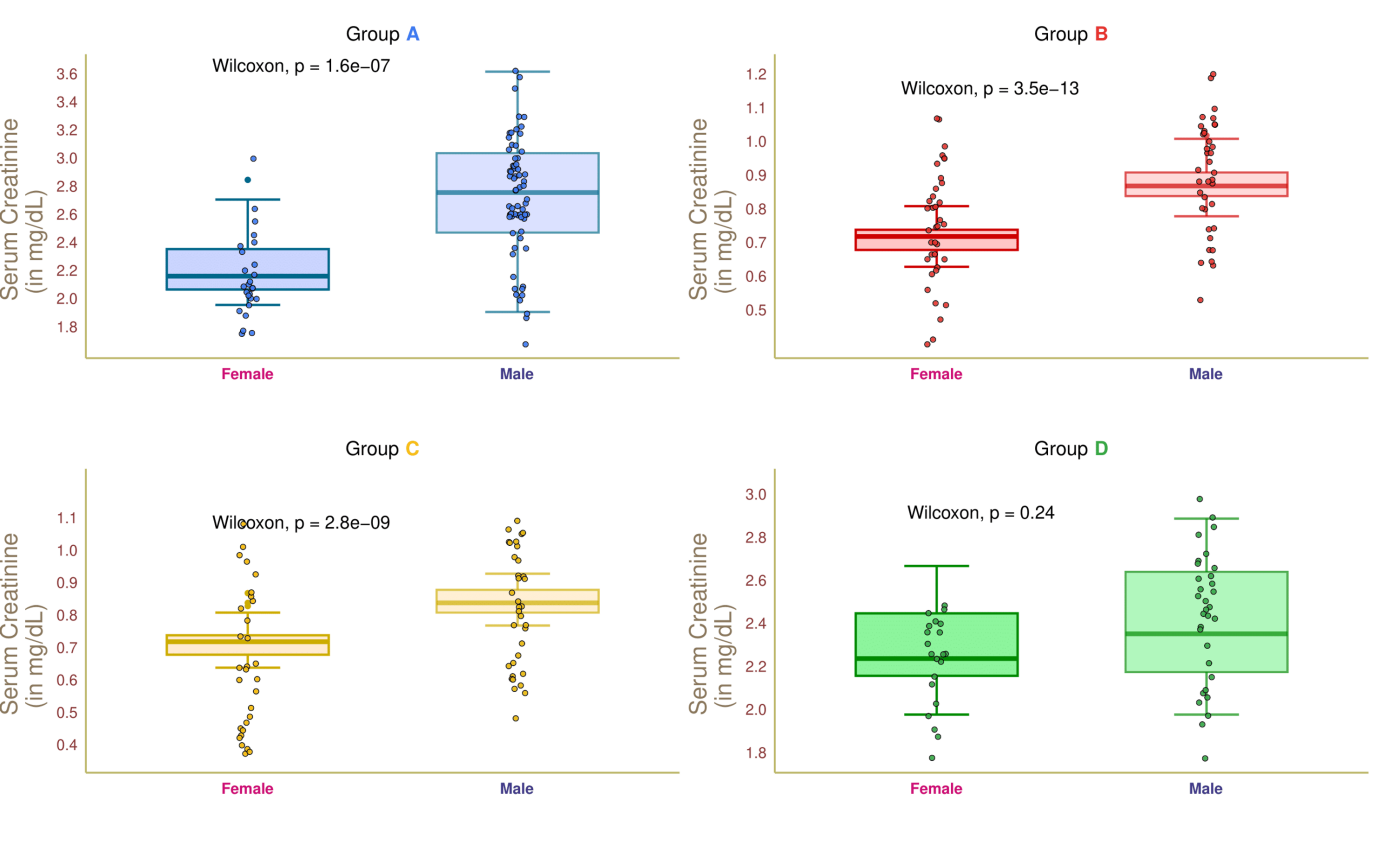
Serum creatinine of female and male participants. The serum creatinine values for the four groups' female and male participants are depicted through the box-whisker and jitter plots. The Wilcoxon test was applied to gauge the differences between female and male participants. Groups A, B, C, and D entailed individuals with diabetic nephropathy, diabetes only, without diabetes and nephropathy, and nephropathy only.

Figure 8 illustrates the serum creatinine values of female and male participants in the four study groups. The median serum creatinine values of group A female and male participants were 2.08 (2.02-2.30) and 2.70 (2.42-3.02) mg/dL, respectively (p < 0.001). The median serum creatinine values of female and male participants in group B were 0.70 (0.68-0.72) and 0.85 (0.82-0.90) mg/dL, respectively (p < 0.001). The median serum creatinine values of group C participants were 0.72 (0.68-0.75) and 0.82 (0.80-0.88) mg/dL, respectively (p < 0.001). The median serum creatinine values of group D participants were 2.22 (2.15-2.45) and 2.32 (2.20-2.62) mg/dL, respectively (p = 0.24). These findings suggested that males had higher creatinine levels as compared to females.

**Figure 9:**
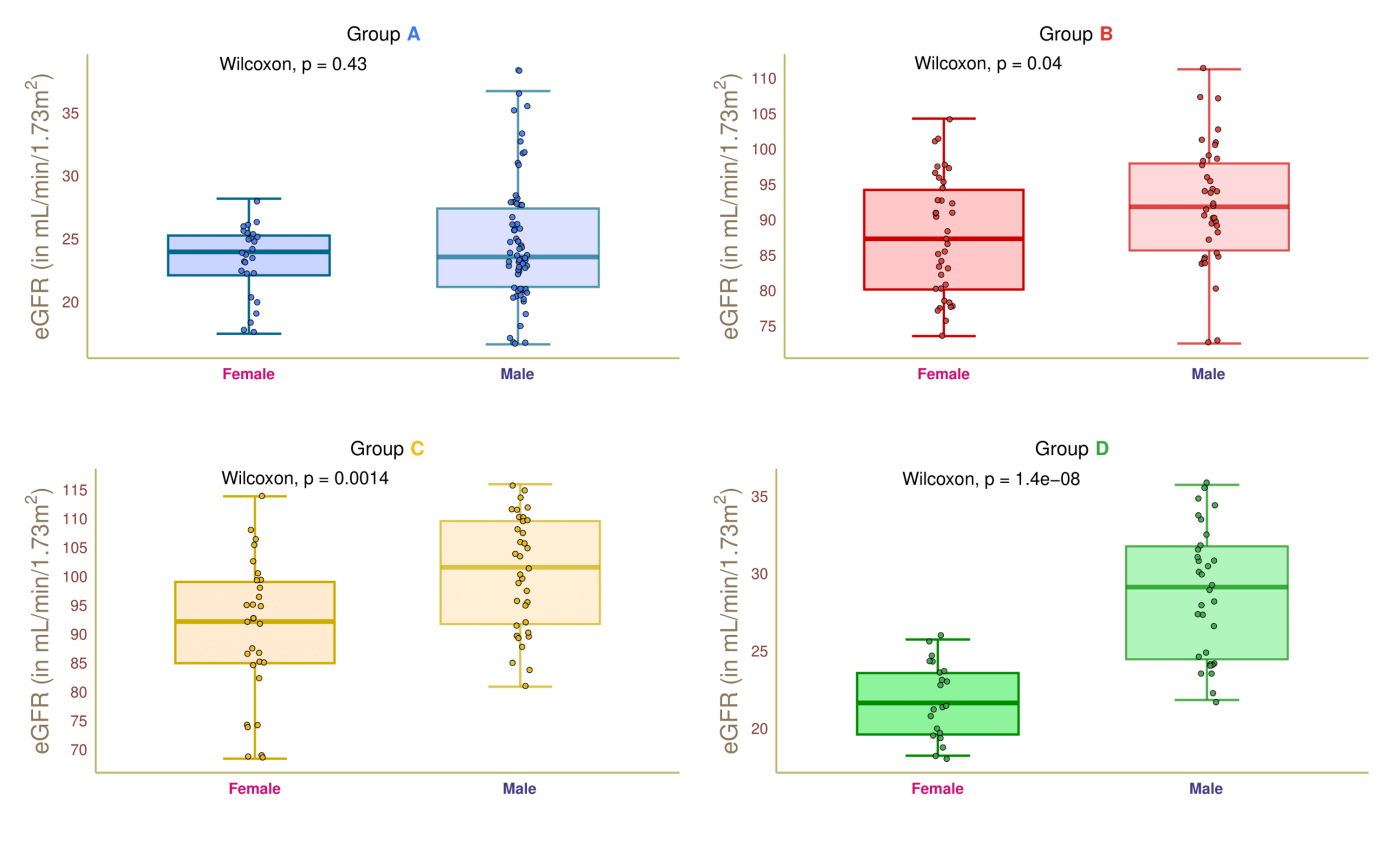
eGFR of female and male participants. The box-whisker and jitter plots depict the eGFR values for the four groups' female and male participants. The Wilcoxon test was applied to gauge the differences between female and male participants. Groups A, B, C, and D entailed individuals with diabetic nephropathy, diabetes only, without diabetes and nephropathy, and nephropathy only. eGFR: estimated glomerular filtration rate.

Figure 9 illustrates the eGFR values of female and male participants in the four study groups. The median eGFR values of group A female and male participants were 24.0 (22.5-25.0) and 23.5 (21.0-27.0) mL/min/1.73 m^2^, respectively (p = 0.43). The median eGFR values of female and male participants of group B were 87.0 (80.5-94.0) and 92.0 (86.5-97.5) mL/min/1.73 m^2^, respectively (p = 0.04). The median eGFR values of group C participants were 92.0 (85.0-98.5) and 101.0 (92.0-108.0) mL/min/1.73 m^2^, respectively (p = 0.001). The median eGFR values of group D participants were 21.5 (19.5-23.5) and 29.0 (25.0-32.0) mL/min/1.73 m^2^, respectively (p < 0.001). These findings suggested that males had a higher eGFR than females, except those with diabetic nephropathy.

In Table 2, we displayed the urine albumin and creatinine values of female and male participants of the four study groups. The median urine albumin values of the participants of the four groups were 4.83 (4.14-5.20), 1.22 (1.11-1.34), 1.04 (0.97-1.09), and 3.77 (3.53-4.12) µg/dL, respectively (p < 0.001). Moreover, the corresponding median urine creatinine values were 24.5 (18.8-30.4), 88.9 (67.9-101.5), 173.6 (154.2-198.6), and 16.9 (14.7-20.1) mg/dL, respectively (p < 0.001). The significant differences yielded from the intergroup comparisons could be attributed to the presence and absence of diabetes mellitus and nephropathy in the corresponding groups. These differences also held good for female and male participants.

**Table 2:** Urine albumin and creatinine values of the study participants. The urine albumin and creatinine values are expressed with the median in the interquartile range (IQR). The intergroup and intragroup differences are weighed with the Kruskal-Wallis and Wilcoxon tests, respectively. Groups A, B, C, and D entailed individuals with diabetic nephropathy, diabetes only, without diabetes and nephropathy, and nephropathy only.

Parameters	Group	Total	Female	Male	p-value
Urine albumin (µg/dL)	A (n = 94; F = 26 & M = 68)	4.83 (4.14-5.20)	3.91 (3.77-4.15)	5.07 (4.65-5.47)	<0.001
	B (n = 75; F = 37 & M = 38)	1.22 (1.11-1.34)	1.13 (1.05-1.22)	1.33 (1.23-1.38)	0.002
	C (n = 65; F = 30 & M = 35)	1.04 (0.97-1.09)	0.96 (0.94-0.98)	1.09 (1.07-1.12)	<0.001
	D (n = 53; F = 21 & M = 32)	3.77 (3.53-4.12)	3.61 (3.53-3.94)	3.81 (3.57-4.20)	0.198
p-value		<0.001	<0.001	<0.001	
Urine creatinine (mg/dL)	A (n = 94; F = 26 & M = 68)	24.5 (18.8-30.4)	24.5 (17.1-25.8)	27.3 (19.5-31.1)	0.031
	B (n = 75; F = 37 & M = 38)	88.9 (67.9-101.5)	89.7 (64.5-104.4)	84.5 (69.1-94.5)	0.507
	C (n = 65; F = 30 & M = 35)	173.6 (154.2-198.6)	154.9 (137.1-198.6)	178.5 (165.5-195.3)	0.026
	D (n = 53; F = 21 & M = 32)	16.9 (14.7-20.1)	16.9 (15.4-21.3)	16.8 (14.6-19.4)	0.742
p-value		<0.001	<0.001	<0.001	

**Figure 10:**
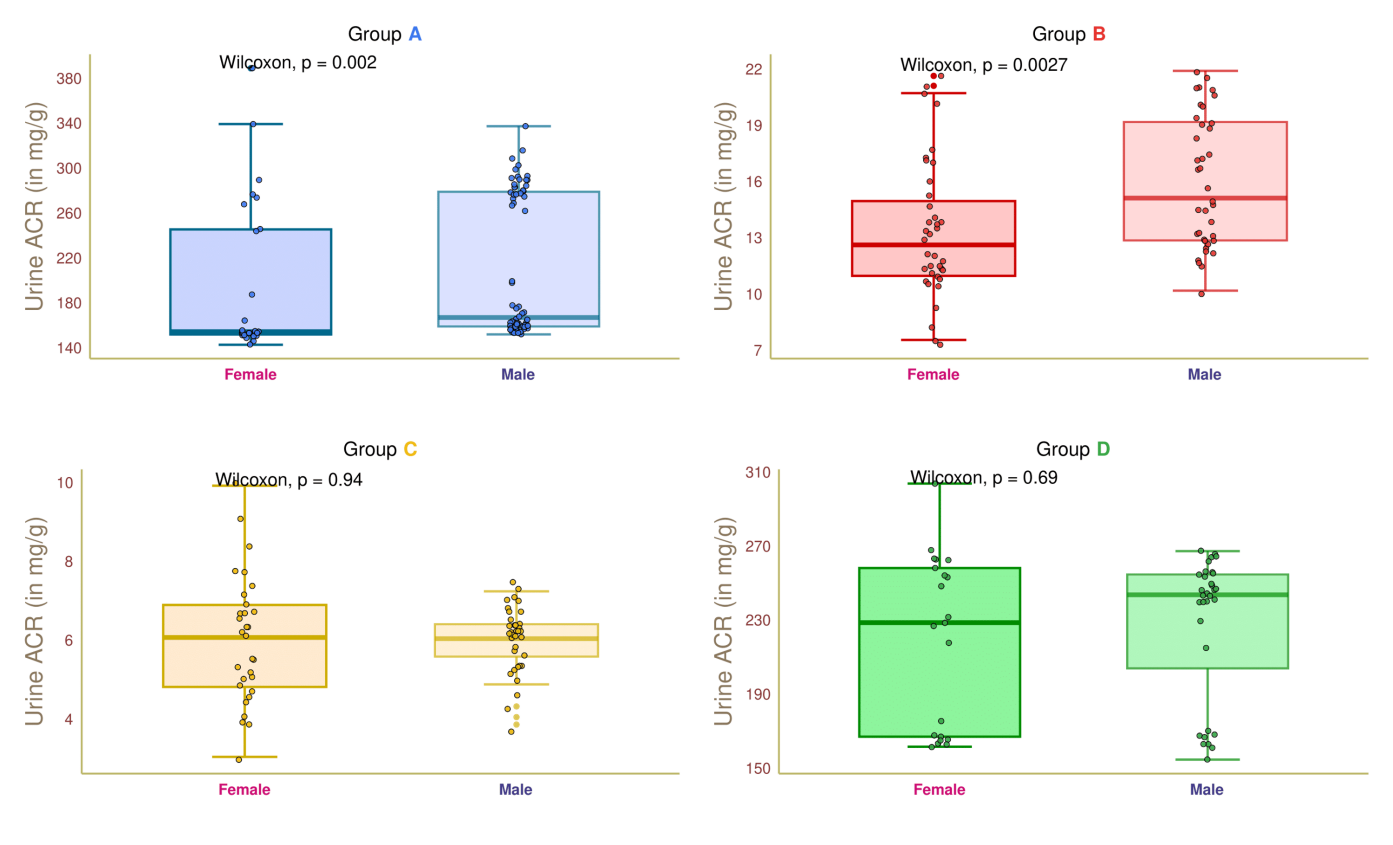
Urine ACR of female and male participants. The box-whisker and jitter plots depict the urine ACR values for the four groups' female and male participants. The Wilcoxon test was applied to gauge the differences among the female and male participants. Groups A, B, C, and D entailed individuals with diabetic nephropathy, diabetes only, without diabetes and nephropathy, and nephropathy only. Urine ACR: ratio of urine albumin to urine creatinine.

Figure 10 illustrates the urine ACR values of female and male participants in the four study groups. The median urine ACR values of group A female and male participants were 155.0 (153.0-243.0) and 264.5 (259.0-276.0) mg/g, respectively (p = 0.002). The median urine ACR values of female and male participants in group B were 12.5 (10.5-15.2) and 15.2 (12.5-19.0) mg/g, respectively (p = 0.003). The median urine ACR values of group C participants were 6.0 (4.8-6.7) and 6.0 (5.5-6.3) mg/g, respectively (p = 0.94). The median urine ACR values of group D participants were 229.5 (166.0-264.5) and 243.0 (204.0-252.0) mg/g, respectively (p = 0.69). These findings suggested that diabetic males had a higher urine ACR than diabetic females.

In Table 3, we displayed the serum sodium and potassium values of female and male participants of the four study groups. The median serum sodium values of the participants of the four groups were 140.1 (137.5-143.9), 140.2 (138.0-142.5), 140.0 (137.9-142.8), and 139.8 (137.3-142.9) mg/dL, respectively (p < 0.001). Moreover, the corresponding median serum potassium values were 4.62 (4.16-4.94), 4.44 (3.95-4.65), 4.48 (4.25-4.66), and 4.91 (3.77-5.10) mg/dL, respectively (p < 0.001). The significant differences yielded from the intergroup comparisons could be attributed to the presence and absence of diabetes mellitus and nephropathy in the corresponding groups. However, the differences between female and male participants were not statistically significant.

**Table 3:** Serum sodium and potassium values of the study participants. The serum sodium and potassium values are expressed with the median in the interquartile range (IQR). The intergroup and intragroup differences are weighed with the Kruskal-Wallis and Wilcoxon tests, respectively. Groups A, B, C, and D entailed individuals with diabetic nephropathy, diabetes only, without diabetes and nephropathy, and nephropathy only.

Parameters	Group	Total	Female	Male	p-value
Serum sodium (mg/dL)	A (n = 94; F = 26 & M = 68)	140.1 (137.5-143.9)	140.3 (134.9-147.1)	140.1 (137.6-143.1)	0.604
	B (n = 75; F = 37 & M = 38)	140.2 (138.0-142.5)	140.3 (138.6-142.6)	139.8 (136.1-142.4)	0.341
	C (n = 65; F = 30 & M = 35)	140.0 (137.9-142.8)	140.2 (138.0-143.2)	139.8 (137.9-141.3)	0.330
	D (n = 53; F = 21 & M = 32)	139.8 (137.3-142.9)	142.2 (138.8-143.2)	139.2 (136.7-141.3)	0.047
p-value		<0.001	0.023	0.017	
Serum potassium (mg/dL)	A (n = 94; F = 26 & M = 68)	4.62 (4.16-4.94)	4.62 (3.93-5.33)	4.62 (4.31-4.89)	0.589
	B (n = 75; F = 37 & M = 38)	4.44 (3.95-4.65)	4.13 (3.96-4.64)	4.57 (3.81-4.68)	0.403
	C (n = 65; F = 30 & M = 35)	4.48 (4.25-4.66)	4.56 (4.32-4.72)	4.31 (4.24-4.60)	0.131
	D (n = 53; F = 21 & M = 32)	4.91 (3.77-5.10)	4.92 (4.51-5.10)	4.90 (3.67-5.07)	0.592
p-value		<0.001	0.008	0.010	

**Figure 11:**
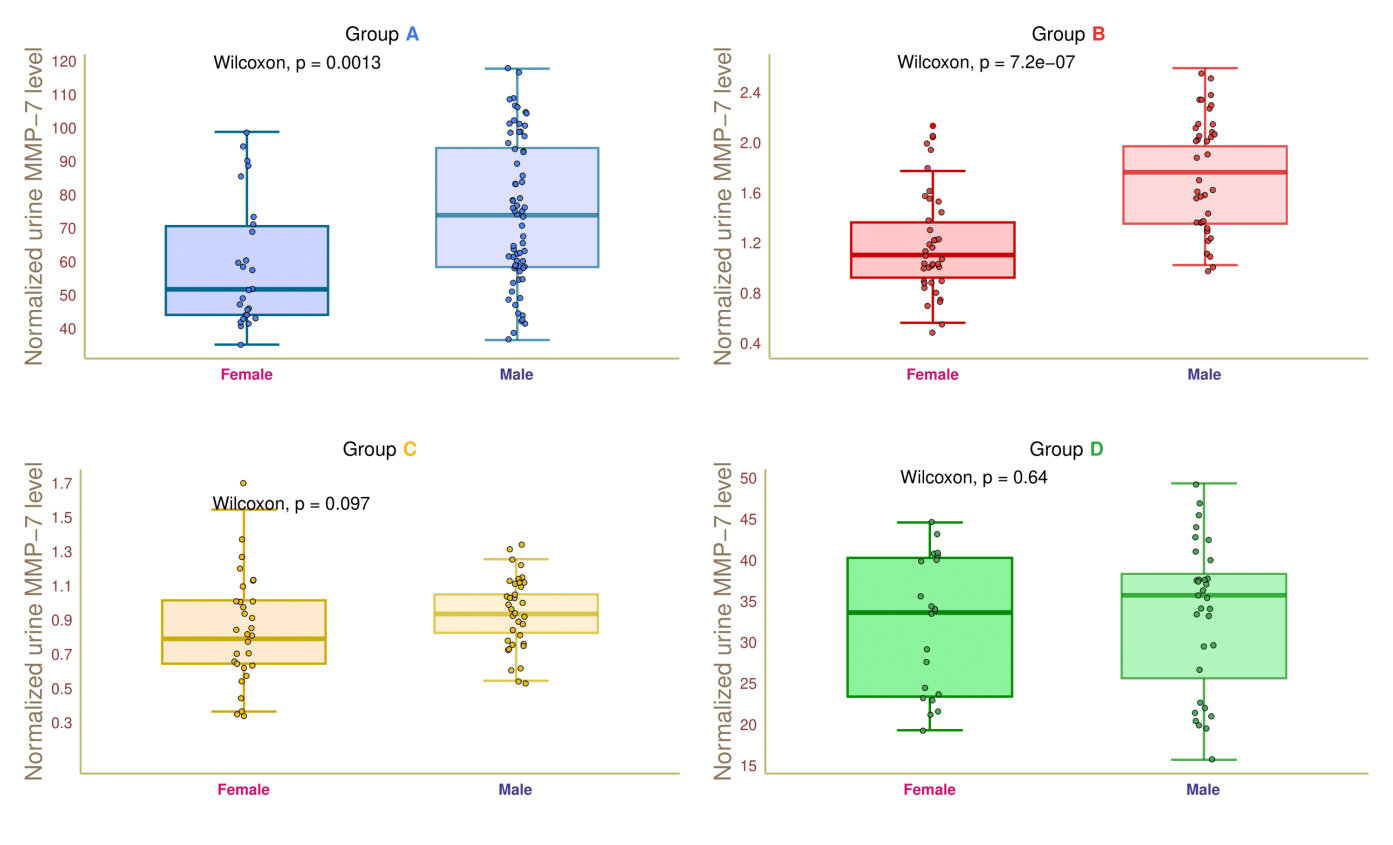
Normalized urine MMP-7 level of female and male participants. The box-whisker and jitter plots depict the normalized urine MMP-7 values for the four groups' female and male participants. The Wilcoxon test was applied to gauge the differences between female and male participants. Groups A, B, C, and D entailed individuals with diabetic nephropathy, diabetes only, without diabetes and nephropathy, and nephropathy only. MMP-7: matrix metalloproteinase 7, normalized urine MMP-7: ratio of urine MMP-7 to creatinine.

Figure 11 illustrates the urine ACR values of female and male participants in the four study groups. The median urine ACR values of group A female and male participants were 51.5 (45.0-70.5) and 72.0 (57.0-93.0) µg/L, respectively (p = 0.001). The median urine ACR values of female and male participants in group B were 1.1 (0.9-1.4) and 1.7 (1.4-1.9) µg/L, respectively (p < 0.001). The median urine ACR values of group C participants were 0.8 (0.6-1.0) and 0.9 (0.8-1.0) µg/L, respectively (p = 0.097). The median urine ACR values of group D participants were 33.0 (23.0-40.0) and 35.0 (26.0-37.5) µg/L, respectively (p = 0.64). These findings suggested that diabetic males had higher urine MMP-7 than diabetic females.

**Figure 12:**
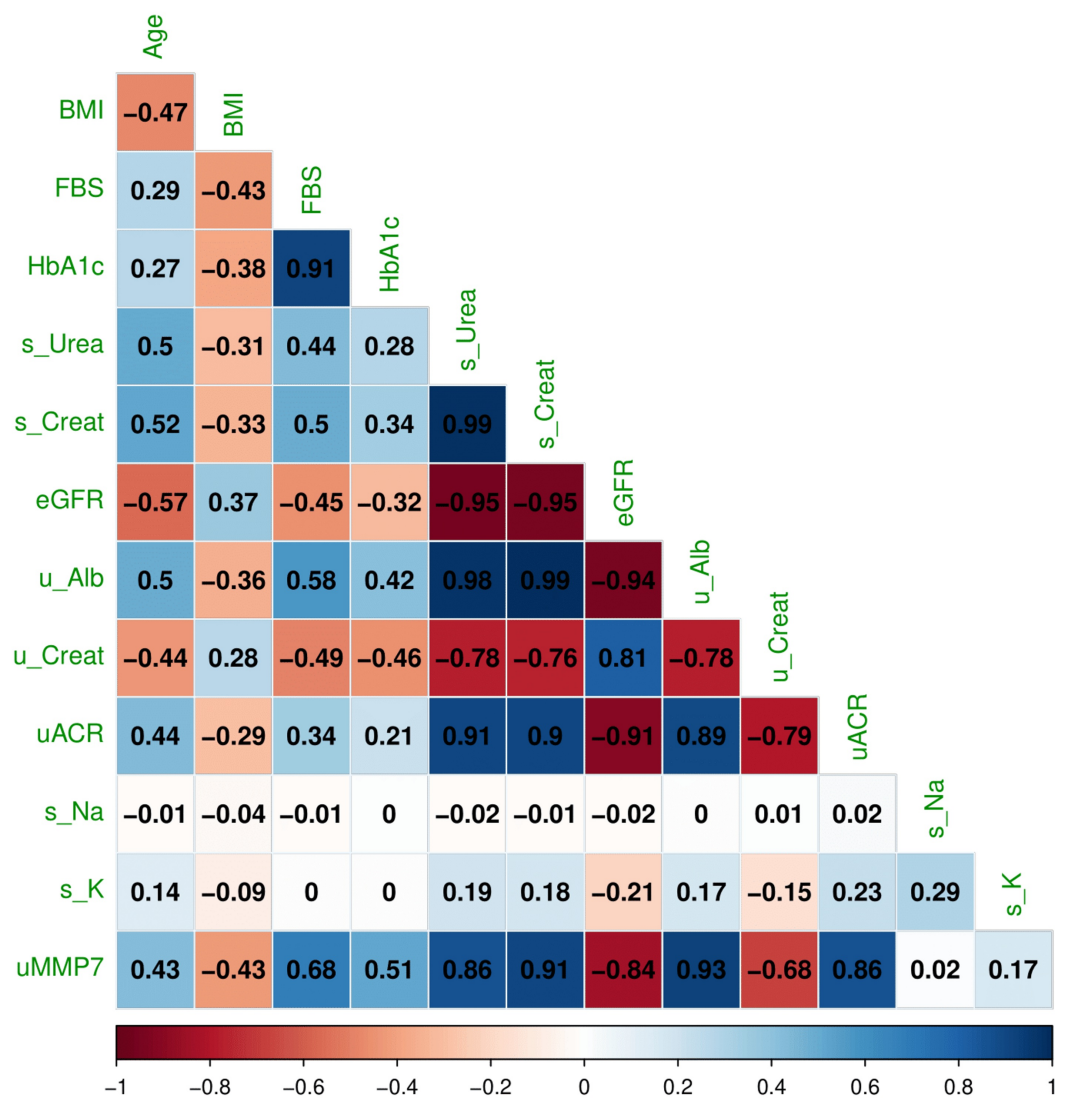
Correlation of various parameters with urine MMP-7 levels. The correlation plot illustrates the degree of association among various parameters. The correlation coefficients range from -1 (strong negative) to +1 (strong positive correlation). BMI: body mass index, FBS: fasting blood sugar, HbA1c: glycosylated hemoglobin, s_Urea: serum urea, s_Crea: serum creatinine, eGFR: estimated glomerular filtration rate, u_Alb: urine albumin, u_Crea: urine creatinine, uACR: ratio of urine albumin to urine creatinine; uMMP-7: ratio of urine MMP-7 (matrix metalloproteinase 7) to urine creatinine.

Figure 12 illustrates the correlation coefficients between various parameters. There were positive correlations between FBS and HbA1c (r = 0.91, 95% CI = 0.89 to 0.93, p < 0.001), serum urea and serum creatinine (r = 0.99, 95% CI = 0.98 to 0.99, p < 0.001), urine albumin and serum creatinine (r = 0.99, 95% CI = 0.99 to 0.99, p < 0.001), and urine ACR and serum creatinine (r = 0.90, 95% CI = 0.88 to 0.92, p < 0.001). There were negative correlations between eGFR and serum creatinine (r = -0.95, 95% CI = -0.96 to -0.93, p < 0.001), eGFR and urine albumin (r = -0.94, 95% CI = -0.95 to -0.93, p < 0.001), urine ACR and eGFR (r = -0.91, 95% CI = -0.92 to -0.88, p < 0.001), and urine creatinine and serum creatinine (r = -0.76, 95% CI = -0.81 to -0.71, p < 0.001). The significant associations with normalized urine MMP-7 were found with urine albumin (r = 0.93, 95% CI = 0.91 to 0.94, p < 0.001), serum creatinine (r = 0.91, 95% CI = 0.88 to 0.93, p < 0.001), serum urea (r = 0.86, 95% CI = 0.83 to 0.89, p < 0.001), urine ACR (r = 0.86, 95% CI = 0.83 to 0.89, p < 0.001), eGFR (r = -0.84, 95% CI = -0.88 to -0.80, p < 0.001), and urine creatinine (r = -0.68, 95% CI = -0.74 to -0.61, p < 0.001). These findings suggested that urine MMP-7 could be considered a screening tool for DKD.

## Discussion

This study evaluated and compared various blood and urine parameters of diabetic and non-diabetic individuals with or without kidney disease. This study investigated the relationship between urinary MMP-7 levels and various renal parameters in diabetic and non-diabetic individuals. We found that diabetic males had higher levels of serum urea, creatinine, and urinary MMP-7 compared to females. These parameters were far-flung because of the diversity of their age, disease durations, glycemic indices, and renal functions. However, the urine ACR and normalized urine MMP-7 harmonized with the eGFR values of the participants, regardless of their age, gender, BMI, HbA1c level, and serum creatinine.

Ninety-four participants in group A had both renal disease (stage IV-V as per the MDRD formula) and T2DM. Group B comprised 75 diabetic individuals without kidney disease. Sixty-five subjects in group C had neither kidney disease nor T2DM. Though they seemed to be healthy, they had either hypertension or dyslipidemia. All the non-diabetic participants in group D had hypertensive kidney disease. We found statistically significant differences in the age and BMI of the participants. This could be attributed to the severity of their ailments. The glycemic indices were similar among female and male subjects in the concerned groups. Diabetic males had higher serum urea and serum creatinine values when juxtaposed with diabetic females. Bhatia et al. [22] found that diabetic males had elevated blood urea nitrogen (BUN) and serum creatinine levels compared to female patients. Our findings concurred with this study. Most of our female participants had a lower eGFR than the males. The research conducted by Webster et al. [7] and Kajiwara et al. [23] corroborated our findings.

Crook et al. investigated the role of genetic polymorphism of the angiotensin-converting enzyme (ACE) gene (I and D alleles for insertion and deletion, respectively) for DKD susceptibility in diabetic individuals [24,25]. Female diabetics with the ACE gene's D allele were more susceptible to DKD progression than males [24,25]. Zambrano-Galvan et al. conducted a study to predict the genetic models of microalbuminuria in patients with DKD. Interleukin-6 (IL-6)'s genetic variant rs1800795 has been linked to a decreased eGFR and the advancement of DKD [26]. It is widely acknowledged that both albuminuria and eGFR are significant attributes of DKD [27]. The microalbuminuric and proteinuric phenotypes are more common in diabetic females and males, respectively [26,27]. Regarding the DKD progression, the findings portrayed that diabetic females, especially those with T2DM in their later years, have a quicker progression and worse outcomes as compared to diabetic males [27]. Our study's findings were consistent with these studies.

After comparing and contrasting the renal parameters of the male and female participants, we discovered that male diabetics had higher serum urea, creatinine, urine albumin, urine creatinine, urine ACR, and normalized urine MMP-7 levels than female diabetics. The normalized urine MMP-7 provided a clearer picture of urinary MMP-7 levels, irrespective of the participants' eGFR values and MDRD staging. In evaluating the predictive value of urinary MMP-7 levels in hypertensive kidney disease, we observed this pattern in our earlier study [18]. The heightened levels of urinary MMP-7 in diabetic individuals could be attributed to their elevated AGEs and dysregulated RAAS and BMP pathways [4,15,16]. We also found a positive correlation between urine ACR and MMP-7 levels (r = 0.86, 95% CI = 0.83 to 0.89, p < 0.001). There was a negative equation between eGFR and urine MMP-7 levels (r = -0.84, 95% CI = -0.88 to -0.80, p < 0.001). Based on these results, urine MMP-7 may serve as a predictive and diagnostic biomarker for DKD assessment prior to changes in serum creatinine and microalbuminuria.

Petra et al., while investigating DKD-associated transcriptomics datasets, discovered a strong association between urine MMP-7 and the progression of DKD [28]. By interacting with the growth factors, cell adhesion molecules, and ECM, MMPs might be involved in crucial biological processes such as angiogenesis, apoptosis, migration, differentiation, and inflammation [29]. MMPs are mainly produced in the kidney by glomerular intrinsic cells and tubular epithelial cells [18,28]. The in-silico study by Petra et al. implied that the production of the CKD-associated peptides may have been primarily caused by MMPs and protein convertases [28].

We are conducting a sensitivity analysis of urine ACR and MMP-7 to diagnose DKD in group A participants. Additionally, we are contrasting the homeostatic model assessment for insulin resistance (HOMA-IR) values with urine MMP-7 values to provide a predictive value for glycemic excursion and insulin sensitivity.

The primary positive aspect of this study was the assessment of individuals with or without T2DM and DKD. The other plus was the correlation of urine MMP-7 levels with various glycemic indices and renal parameters. A couple of points in our study could have been refined. First, we could have compared these values on a long-term basis. Second, we did not evaluate the severity of the comorbidities and the role of concomitant medications. Third, numerous etiologic causes for kidney disease affect various aspects of quality of life. In real-world settings, it might be tricky to verify each of these points in a patient on long-term treatment for T2DM, hypertension, or DKD.

## Conclusions

This study evaluated the role of urinary MMP-7 as a preliminary indicator of kidney disease in individuals with T2DM. We compared the renal parameters like serum urea, creatinine, sodium, potassium, urine albumin, creatinine, urine ACR, eGFR, and urine MMP-7 levels among the participants with or without T2DM and kidney disease. We also assessed glycemic parameters like FBS and HbA_1c_. We found that diabetic males had higher serum urea, serum creatinine, eGFR, urine ACR, and urine MMP-7 levels than diabetic females. The urine MMP-7 level was strongly correlated to serum creatinine, eGFR, and ACR. Further studies with longer study durations and larger sample sizes are warranted to evaluate the long-term changes of urine MMP-7 in patients with T2DM and DKD.
